# Rapid and Visual Detection of *Heterodera schachtii* Using Recombinase Polymerase Amplification Combined with Cas12a-Mediated Technology

**DOI:** 10.3390/ijms222212577

**Published:** 2021-11-22

**Authors:** Ke Yao, Deliang Peng, Chen Jiang, Wei Zhao, Guangkuo Li, Wenkun Huang, Lingan Kong, Haifeng Gao, Jingwu Zheng, Huan Peng

**Affiliations:** 1 Institute of Biotechnology, College of Agriculture and Biotechnology, Zhejiang University, Hangzhou 310058, China; 17794539779@163.com; 2 State Key Laboratory for Biology of Plant Diseases and Insect Pests, Institute of Plant Protection, Chinese Academy of Agricultural Sciences, Beijing 100089, China; pengdeliang@caas.cn (D.P.); 18684759602@163.com (C.J.); zw2460018926@126.com (W.Z.); wkhuang2002@163.com (W.H.); lakong@ippcaas.cn (L.K.); 3 Key Laboratory of Integrated Pest Management on Crop in Northwestern Oasis, Ministry of Agriculture, Institute of Plant Protection, Xinjiang Academy of Agricultural Sciences, Scientific Observing and Experimental Station of Korla, Urumqi 830091, China; lgk0808@163.com (G.L.); ghf20044666@163.com (H.G.)

**Keywords:** recombinase polymerase amplification (RPA), sugar beet cyst nematode, *Heterodera schachtii*, lateral flow dipstick (LFD), Cas12a

## Abstract

*Heterodera schachtii* is a well-known cyst nematode that causes serious economic losses in sugar beet production every year. Rapid and visual detection of *H. schachtii* is essential for more effective prevention and control. In this study, a species-specific recombinase polymerase amplification (RPA) primer was designed from a specific *H. schachtii* sequence-characterized amplified region (SCAR) marker. A band was obtained in reactions with DNA from *H. schachtii*, but absent from nontarget cyst nematodes. The RPA results could be observed by the naked eye, using a lateral flow dipstick (LFD). Moreover, we combined CRISPR technology with RPA to identify positive samples by fluorescence detection. Sensitivity analysis indicated that 10^−4^ single cysts and single females, 4^−3^ single second-stage juveniles, and a 0.001 ng genomic DNA template could be detected. The sensitivity of the RPA method for *H. schachtii* detection is not only higher than that of PCR and qPCR, but can also provide results in <1 h. Consequently, the RPA assay is a practical and useful diagnostic tool for early diagnosis of plant tissues infested by *H. schachtii*. Sugar beet nematodes were successfully detected in seven of 15 field sugar beet root samples using the RPA assay. These results were consistent with those achieved by conventional PCR, indicating 100% accuracy of the RPA assay in field samples. The RPA assay developed in the present study has the potential for use in the direct detection of *H. schachtii* infestation in the field.

## 1. Introduction

More than 4100 plant-parasitic nematode (PPN) species have been described [1], and the estimated annual loss caused by PPNs is more than 100 billion USD, mainly attributable to cyst nematodes (*Heterodera* spp. and *Globodera* spp.) and root-knot nematodes (*Meloidogyne* spp.) [2]. Among cyst nematodes, the sugar beet cyst nematode (SBCN), *Heterodera schachtii*, is an important plant parasite that greatly impacts sugar beet cultivation worldwide [3]. *H. schachtii* belongs to the *H.* *schachtii* sensu stricto group, which also includes *H. glycines*, *H. betae*, *H. ciceri*, *H. daverti*, *H. medicaginis*, and *H. trifolii* [4]. *H. schachtii* and its related species are usually distinguished by cyst morphology, which requires significant professional knowledge; moreover, the identification process is time-consuming and laborious. To enhance accuracy and sensitivity, molecular detection techniques have developed rapidly in recent years. A series of DNA-based detection methods have been developed for the identification of SBCN. Polymerase chain reaction (PCR)-random amplified polymorphic DNA (RAPD) and amplified fragment length polymorphism (AFLP) have been used to determine the genetic diversity of SBCN populations [5,6].

Other methods include detection by PCR internal transcribed spacer (ITS) restriction fragment length polymorphism (RFLP) [7], designing a specific primer, SHF6, that was used in combination with the universal primer, rDNA2, downstream of the ITS, to amplify a specific 255 bp fragment from SBCN [8]. Subsequently, the specific primer was modified and used in a multispecies-specific quantitative real-time PCR (qPCR) method to identify *H. schachtii, G. pallida*, and *G. rostochiensis* [9]. Recently, Baidoo and Yan [10] developed a real-time PCR assay based on sequences of the nematode effector, CLAVATA, for direct identification and quantification *H. glycines* and *H. schachtii* in soil.

In recent years, several new technologies have been introduced for the rapid detection of plant diseases, including fungi and nematodes, under field conditions, such as loop-mediated isothermal amplification (LAMP) [11,12] and recombinant enzyme polymerase amplification (RPA) [13]. RPA is an in vitro isothermal nucleic acid amplification technique that can be performed at a constant temperature of 37 °C to 42 °C, and the reaction can be completed within 20 min [14]. As with PCR, the size of the amplicon produced by RPA is determined by primer binding. RPA has the advantages of high efficiency, fast amplification, and constant operating temperature [15]. Using a lateral flow dipstick (LFD) strip, RPA amplicons can be observed directly with the naked eye, making it suitable for application in the field [13]. To date, RPA has been successfully used to detect a variety of plant parasitic nematodes [16], including *Meloidogyne* spp., such as *M. incognita*, *M. arenaria*, *M. javanica*, *M. enterolobii* [17], *M. javanica* [18], and *M. enterolobii* [19]. In addition, Cha et al. reported the application of RPA for detecting *Bursaphelenchus xylophilus* [20,21]. Furthermore, a method was recently developed for rapid detection of human diseases by combining programmable nucleotide targets carried by Cas proteins (such as Cas12a and Cas13a) with nucleic acid rapid lateral flow immunoassay (NALFIA) [22,23,24]. Cas12a was combined with the RPA technique based on the nonspecific single-stranded DNA (ssDNA) cleavage characteristics of Cas12a, with sample detection completed by the targeted binding of RPA products to guide CRISPR RNA (CrRNA) [25,26]. This combined method is a simple, relatively cost-effective, rapid, specific, and highly sensitive detection system that does not require special technical expertise or auxiliary equipment, and that has higher sensitivity. For example, an RPA/CRISPR nucleic-acid-based assay was developed for early rabies diagnosis by detecting viral RNA shedding in the cerebrospinal fluid (CSF) of rats [27]. This technology has been used to develop an isothermal CRISPR-based diagnostic system for COVID-19, with near single-copy sensitivity [28]. In addition, RPA/CRISPR has been applied for the detection of plant-pathogenic bacteria. Kang et al. [29] developed a method to detect the specific sequence of *Magnaporthe oryzae Triticum* (MoT) in infected wheat plants by combining the target gene-dependent activation of Cas12a ssDNase with RPA and NALFIA. However, the use of RPA/CRISPR for plant nematode detection has yet to be reported.

In this study, we designed RPA primers on the basis of a specific SCAR fragment of *H. schachtii* and developed a simple, rapid, and visual assay for the detection of *H. schachtii* in infected roots and soil samples using a combination of the RPA method, LFD strips, and CRISPR technology. This method is potentially valuable for direct detection of *H.* *schachtii* in field samples.

## 2. Results

### 2.1. Primer Design

**Figure 1:**
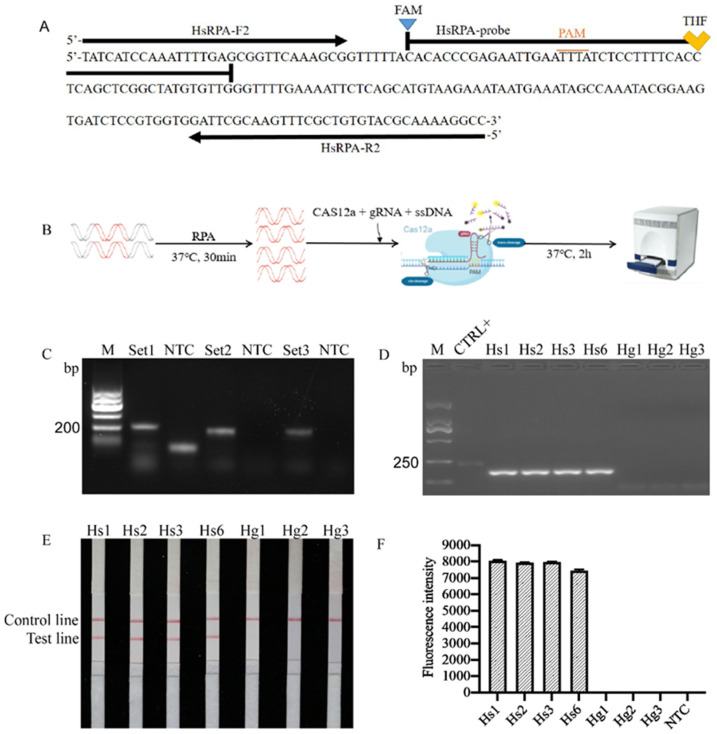
Screening of RPA primers for *H. schachtii*. (**A**) Partial nucleotide sequence of *H. schachtii*, showing the designed primers and probes. The position and amplification direction of the primers and probes are marked with black arrows. (**B**) Cas12a-mediated method of detection of DNA samples containing *H. schachtii* sequence. (**C**) Amplification bands generated using three pairs of RPA primers. (**D**) Specific amplification with selected primers was detected by agarose gel electrophoresis. (**E**) Specific amplification with selected primers detected using LFD test strips. (**F**) Specific amplification with selected primers detected using the RPA/Cas12a method. PAM, protospacer adjacent motif site (TTTA) for Cas12a recognition; CTRL+, the positive RPA control provided by the kit; M, D2000 marker; NTC, no template was added as a negative control. The presence of two lines indicates a positive result.

To estimate the specificity of the sequences of RAPD fragments (GenBank accession No. MW854319, accessed on 1 April 2021), the sequence was tested against NR database and published nematode whole-genome shotgun contig databases (taxid: 6231) on the NCBI website. The blast results indicated that the sequence was similar to *H. schachtii* genomic sequences (*JAHGVF010000211.1*) and *H. glycines* genomic DNA sequences (*VAPQ01000257.1*) with 99.24% and 86.35% identity, respectively. The sequences also had homology (identity values <73%) to genomic sequences of *G. pallida* (*CBXT010012390.1*), *G. ellingtonae* (*MEIZ01000021.1*), *G. rostochiensis* (*JAEVLO010000032.1*), *Meloidogyne incognita* (*RCFL01002996.1*), *M. arenaria* (*QEUI01000363.1*), *M. javanica* (*RCFK01013271.1*), and *M. enterolobii* (*CAJEWN010003051.1*) with low query cover (<29%). The alignment results are shown in Figure S1; three sets of RPA primers and probes were designed on the basis of specific RAPD sequences (Table 1). The results of Primer-Blast searches showed that the primer pairs were specific to the SCAR template, as no other targets were found in the Nr database. Amplification results using the three pairs of primers are shown in Figure 1. Considering primer dimers and nonspecific reaction products, one primer set (HsRPA-F2 and HsRPA-R2) was selected for use in subsequent experiments (Figure 1A). *H. schachtii* and *H. glycines*, both members of the *H.* *schachtii* sensu stricto group, were used as templates to evaluate whether the selected RPA primers were specific for *H. schachtii*, and positive results were only obtained with *H. schachtii* samples (Figure 1D–F).

### 2.2. Detection and Confirmation of RPA Products

RPA products were separated by 2% agarose gel electrophoresis (Figure 1D). To eliminate false-positive interference, RPA reaction products were also evaluated using LFDs. Samples with positive amplification results generated both control and test lines in LFDs, whereas negative control samples displayed only the control line (Figure 1E). Furthermore, we also increased the accuracy of detection by using RPA combined with Cas12a technology, and the fluorescence was detected only in the system containing *H. schachtii* gDNA (Figure 1B,F).

### 2.3. Specificity Test

**Figure 2:**
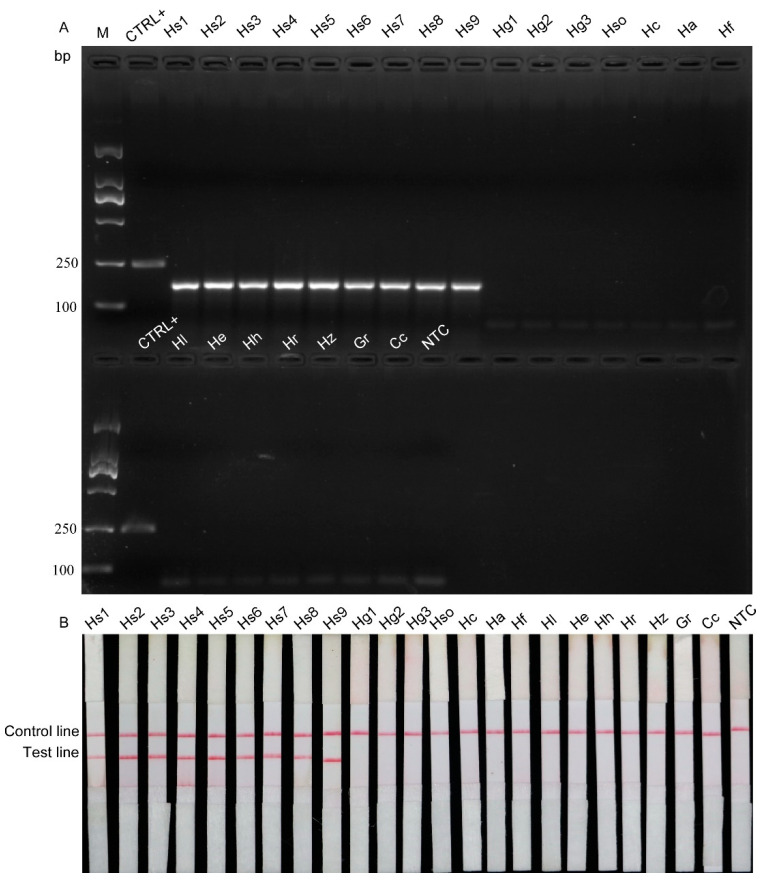
Verification of assay specificity for *H. schachtii*. (**A**) Agarose gel electrophoresis results. (**B**) LFD test strip results. CTRL+, the positive RPA control provided by the kit; M, D2000 marker; NTC, no template was added as a negative control. The presence of two lines indicates a positive result.

The specificity of the RPA method was determined using nine populations of *H. schachtii* and 14 other cyst nematode species (Table 2). Positive bands were observed in reactions containing *H. schachtii* gDNA, but not in those containing DNA from other nematode species (Figure 2A). Moreover, the RPA products were tested using LFD strips (Figure 2B). Two lines, including a test line and control line, were visible in samples containing *H. schachtii* DNA template, while the test line was absent when DNA from another cyst nematode or sterile H_2_O were used as template. These results suggest that RPA combined with LFD strips can distinguish *H. schachtii* from *H. glycines* and other closely related cyst nematode species.

### 2.4. Sensitivity Test

**Figure 3:**
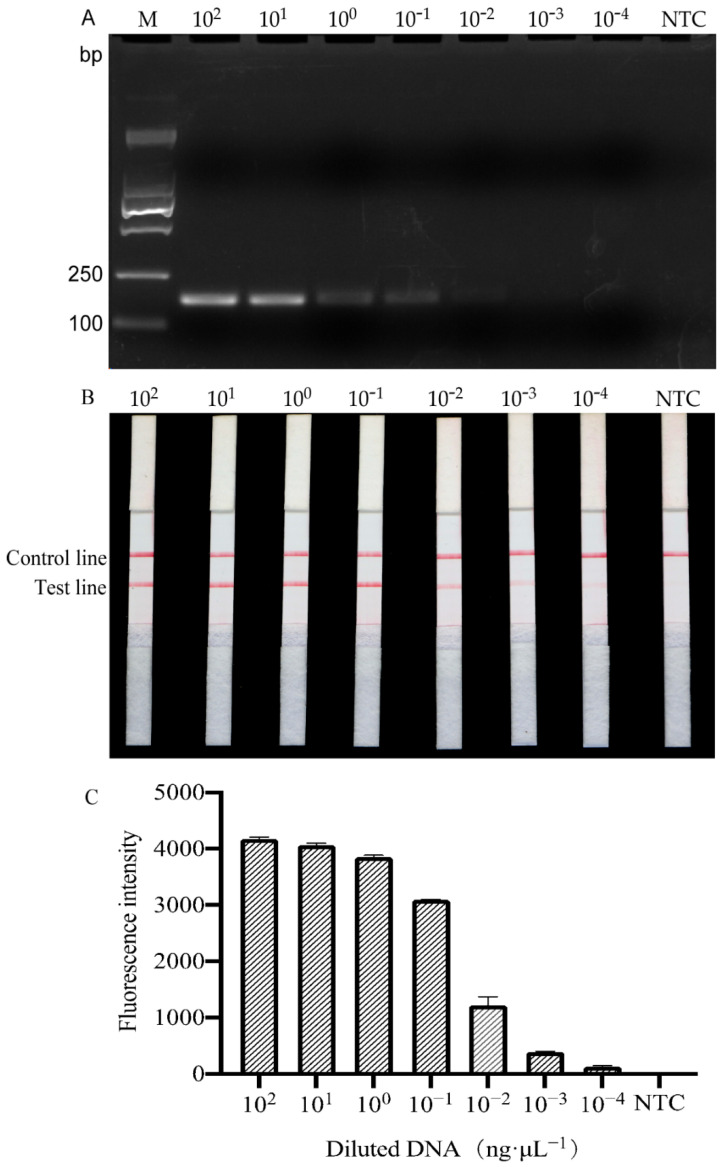
DNA concentration gradient sensitivity test of the RPA system. (**A**) Agarose gel electrophoresis results. (**B**) LFD test strip results. (**C**) Fluorescence was measured using the RPA/Cas12a method. M, D2000 marker; NTC, no template was added as a negative control. The presence of two lines indicates a positive result.

**Figure 4:**
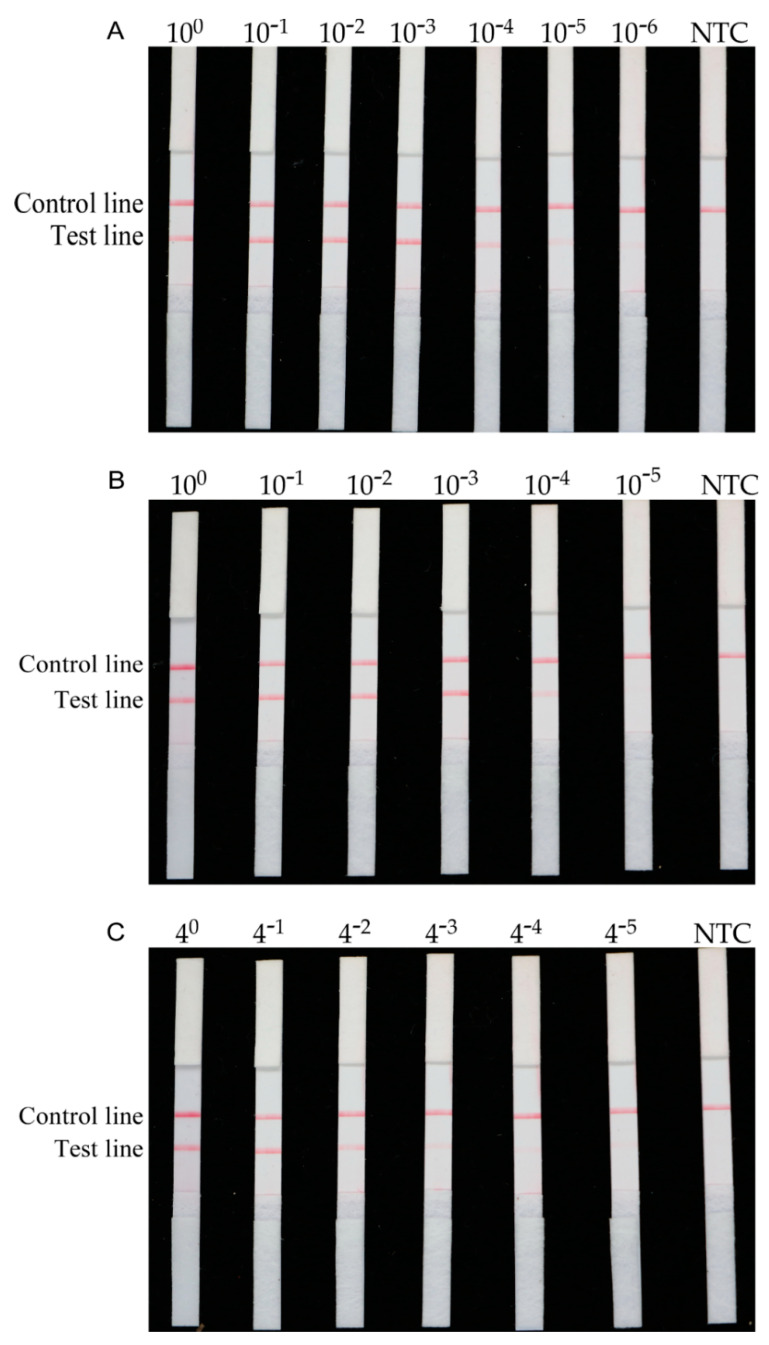
Molecular verification of sensitivity of the RPA system using gradient-diluted *H. schachtii*. (**A**) Gradient-diluted cyst sample. (**B**) Gradient-diluted female sample. (**C**) Gradient-diluted second-stage juvenile sample. NTC, no template was added as a negative control. The presence of two lines indicates a positive result.

Next, quantitative DNA templates were used to evaluate the sensitivity of the RPA method. The results of electrophoresis showed that a visible band was amplified when the *H. schachtii* DNA concentration was as low as 0.1 ng/μL (Figure 3A). The LFD test strip and RPA combined with Cas12a were more sensitive than electrophoresis, with a sensitivity of 0.001 ng/μL (Figure 3B,C). Furthermore, serial dilutions of *H. schachtii* DNA isolated from a single J2, cyst, and female nematode specimens were used as RPA templates, and the results indicated that the RPA method developed in this study could detect 10^−4^ dilutions of *H. schachtii* DNA from a single cyst and a single female, and a 1/64 dilution of DNA from a single J2 (Figure 4).

### 2.5. Direct Detection of Heterodera schachtii in Artificially Inoculated Roots

**Figure 5:**
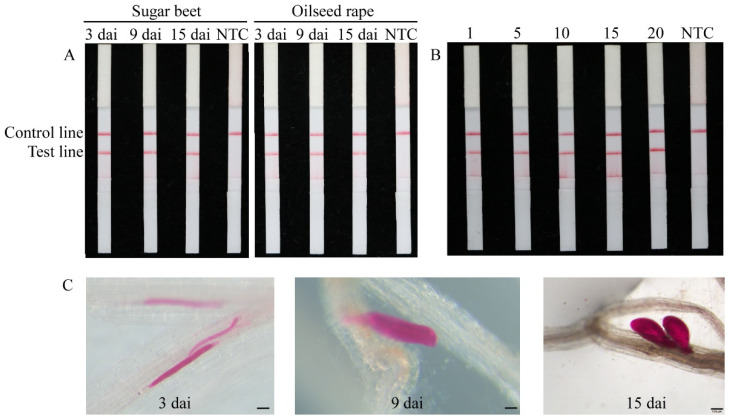
Application of the RPA system for direct detection of *H. schachtii* in soil and plant samples. (**A**) *H. schachtii* in sugar beet and oilseed rape roots. (**B**) *H. schachtii* in soil. (**C**) Morphology of *H. schachtii* in sugar beet roots on various days after inoculation, bar = 100 µm. NTC, no template was added as a negative control. The presence of two lines, including a control line and test line, indicates a positive result.

Two host plants, sugar beet and oilseed rape, artificially inoculated with *H. schachtii,* were used to assess the applicability of the RPA assay. DNA was extracted from plant roots 3, 9, and 15 days after inoculation (dai). RPA/LFD analysis accurately identified one J2 per 10 g of soil (Figure 5A). In addition, the results of RPA/LFD detection showed that all root samples had positive bands (Figure 5B). Results from analysis of uninoculated roots and autoclaved soil samples were negative.

### 2.6. Detection of Heterodera schachtii in Natural Field Samples

**Figure 6:**
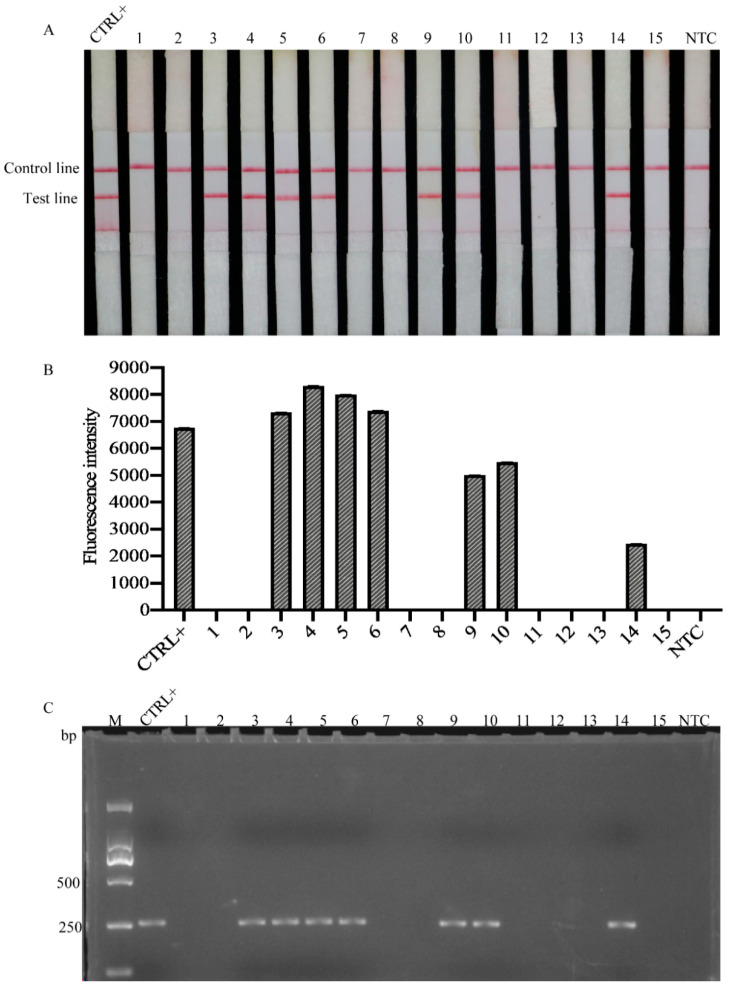
Detection of *H. schachtii* in natural field samples using the RPA assay and conventional PCR. (**A**) LFD test strip results. (**B**) RPA/CRISPR results. (**C**) Conventional PCR results. M, D2000 marker; CTRL+, positive control containing *H. schachtii* DNA; NTC, no template was added as a negative control. The presence of two lines indicates a positive result.

A field experiment was conducted using 15 field sugar beet root samples from Xinjiang, Inner Mongolia, and Hebei Provinces, to evaluate the practicality of the RPA method (Table 3). Positive results were observed in seven of the 15 samples (Xinyuan County and Emin County, Xinjiang Province and Zhangbei County, Hebei Province) using both RPA/LFD and RPA/CRISPR technologies, while the other eight samples were negative (Figure 6A,B). The results of conventional PCR were consistent with those of RPA analysis (Figure 6C).

## 3. Discussion

The sugar beet cyst nematode, *H. schachtii*, poses a serious economic threat to sugar beet production worldwide [41,42]. Unfortunately, this species was reported recently in Xinyuan County, Xinjiang Province of China [43]. Therefore, there is an urgent need to develop more effective control measures. Rapid and accurate identification of *H. schachtii* is essential for the development of more effective management of this species. Previous detection of *H. schachtii* largely depended on PCR technology, using specific primers [8]. However, this method has limitations, as it requires a PCR instrument and a relatively long time (4–8 h) to obtain a diagnostic result, and it is less suitable for field detections. Unlike LAMP technology, the application of RPA technology overcomes the limitations of high reaction temperature conditions (60–65 °C) [29]. In addition, RPA is suitable for use in small local laboratories, because it does not rely on expensive instruments and complex testing steps to obtain the results, which can be observed by the naked eye using an LFD strip. Many studies have confirmed that the sensitivity of the RPA assay is higher than that of conventional PCR [44]. In this study, according to RPA primer design principles, we designed RPA primers which were specific on NCBI Primer-Blast analysis based on the specific RPAD sequences with OPA06 primer (GenBank accession No. MW854319, accessed on 1 April 2021) and used them to successfully detect *H. schachtii*.

The primers (HsRPA-F2 and HsRPA-R2) used in this study are located in the genomic DNA sequence where differences exist between *H. schachtii* and other plant nematodes (Figure S1) to ensure the specificity of detection by the RPA assay. In addition, to estimate the specificity of our RPA assay, a variety of cyst nematode species, including *Heterodera*, *Globodera*, and *Cactodera* spp., were tested in the present study. Positive results were obtained only using nine populations of *H. schachtii,* but were not generated from 14 closely related *Heterodera* species, including *H. glycines*, *H. sojae*, *H. cruciferae*, *H. avenae*, *H. filipjevi*, *H. latinpones*, *H. elachista*, *H. humuli*, *H. ripae*, *H. zeae*, *G.* *rostochiensis*, and *Cactodera cacti*, suggesting high specificity (Figure 2). Radice et al. reported that it is extremely difficult to differentiate *H. schachtii* and *H. glycines,* as they are very similar and closely related species that diverged from a common ancestor [45]. Our results demonstrate that *H. schachtii* can be distinguished from *H. glycines* using the RPA method, indicating the high specificity of this technique. *H. schachtii* is a member of the *H. schachtii* group, which includes more than 10 species [4]. Hence, to avoid the risk of misdetection, more nematodes and other related species should be analyzed in the future.

In a previous report, Amiri et al. detected 0.6 ng of *H. schachtii* genomic DNA or 1/1000 cysts using a PCR species-specific primer [8]. Furthermore, a more rapid method for the quantification of *H. schachtii* using real-time PCR with SYBR Green I-based dye and a detection sensitivity <100% was developed for samples containing <5 *H. schachtii* J2s [9]. In our study, the results of sensitivity testing showed that the RPA assay could detect *H. schachtii* in 10^−4^ cysts, 10^−4^ females, 4^−3^ of J2, and 0.001 ng of genomic DNA (Figure 4). Hence, the sensitivity of the RPA method is higher than that of PCR-based detection methods. Traditional detection methods include PCR and qPCR, where PCR is a primary DNA detection method with amplified DNA visualized by gel electrophoresis techniques [46] and quantitative PCR is an updated PCR method. However, these assays are not suitable for poorly equipped laboratories or field diagnosis [47]. The sensitivity of the RPA test is not only higher than that of PCR and qPCR, but can also provide results in <1 h.

The RPA/LFD method developed here has advantages in not requiring expensive equipment and providing rapid results. Furthermore, the results can be observed by the naked eye, making the method suitable for use in the field. In addition, RPA technology can be used to identify target nematodes in plant roots and soil samples. The combination of Cas12a and RPA technology improves the speed and sensitivity of detection [25]. In this study, Cas12a was chosen because it has greater reported target specificity than Cas9 nuclease [48]. We used RPA binding to the Cas12a protein to enhance the reliability and sensitivity of detection, and we then measured the fluorescence values to determine whether samples contained the target. Our data demonstrate that the RPA/Cas12a method has higher sensitivity than that of electrophoresis, and that the sensitivity of the RPA/LFD and RPA/Cas12a methods is similar. The sensitivity of detection reported here was also higher than that for detection of *M. hapla* by RPA/LFD, which was 1/10 for a second-stage juvenile and 1/1000 for a female [49]. In field testing, the RPA/Cas12a method can be replaced with a handheld fluorescence reader. Therefore, this assay requires only a portable thermostatic heater and handheld fluorescence reader to complete sample detection. In the early stage of cyst nematode infection, there is no obvious damage to plant roots. Therefore, the optimal period for implementation of prevention and treatment measures is often missed because of difficulties in diagnosis. In this study, we generated positive results from infected sugar beets and oilseed rape roots at 3, 9, and 15 dai, demonstrating that *H. schachtii* can be detected at different stages in the roots of oilseed rape and sugar beet plant (Figure 5A,C). In addition, we could accurately identify one J2 per 10 g of soil (Figure 5B).

SBCN is an important quarantine nematode in China and globally. Unfortunately, it has been found in Xinyuan County Xinjiang Province of China [43]. To further detect the occurrence and distribution of sugar beet cyst nematode in China, 10 field sugar beet samples were collected from the counties surrounding Xinyuan County; in addition, five other samples were collected from Inner Mongolia and Hubei Provinces, the main sugar beet-growing areas in China. In this study, RPA was performed by directly extracting DNA from natural root specimens, and seven out of 15 natural root samples were positive (Figure 6, Table 3). These results were consistent with those of traditional PCR results, verifying the reliability of the RPA method for the detection of *H. schachtii* in natural root samples, indicating that, in addition to the distribution of sugar beet cyst nematodes in Xinyuan County, Xinjiang, the nematodes are also present in Emin County (Xinjiang Province) and Zhangbei County (Hebei Province). Xinjiang province is one of the main sugar beet-planting areas in China, and *H. schachtii* is an economically important plant-parasitic nematode that limits sugar beet cultivation worldwide [2]. Thus, there is an urgent need for the development of a rapid and accurate diagnostic assay that can be used for direct detection of the nematode in the field.

In the present study, we developed a rapid and accurate RPA assay for *H. schachtii* detection. The method has high specificity and sensitivity, and it facilitates rapid, accurate, and direct detection of *H. schachtii* from infected plant tissue. This assay is potentially useful for monitoring and management of *H. schachtii* in the field.

## 4. Materials and Methods

### 4.1. Nematode Populations

In this study, we analyzed nine populations of *H. schachtii* from Xinjiang Province (China), Germany, Belgium, and Turkey, three populations of *H. glycines*, and 11 other cyst nematode species (Table 2). All nematode populations were confirmed by morphological examination and ITS ribosomal DNA (rDNA) sequence analysis. Second-stage *H. schachtii* juveniles (J2s) were hatched by exposure to 3 mM ZnCl_2_ solution at 25 °C [50].

### 4.2. DNA Extraction

A single nematode was placed into 10 µL of sterilized distilled water in a PCR tube and frozen in liquid nitrogen. Then, the nematode was crushed with a sterilized glass rod. PCR buffer (10 × 8 µL; Takara-Bio, Shiga, Japan) and 2 µL of Proteinase K solution (600 µg/mL, Roche, Basel, Switzerland) were added to the sample in the PCR tube, and then the mixture was frozen at −80 °C for 2 h. The PCR tube was then incubated at 65 °C for 1.5 h, followed by incubation at 95 °C for 10 min, and it was finally centrifuged at 10,000× *g* for 1 min. The supernatant DNA suspension was stored at −20 °C. Pure genomic DNA was also isolated from 10,000 *H. schachtii* J2s; nematodes were frozen in liquid nitrogen and ground with a mortar and pestle until they were completely homogenized. DNA was extracted from the resulting macerate using a DNeasy Blood and Tissue Kit (Qiagen, Hilden, Germany). For DNA extraction from plant root samples, approximately 0.2 g of root was cut, frozen in liquid nitrogen, and ground to powder; then, genomic DNA was obtained by adding solvents from a Universal Genomic DNA Extraction Kit (Takara-Bio, Shiga, Japan), according to the manufacturer’s instructions. To isolate nematode DNA from artificially infested soil and natural field soil, soil samples were mixed, and a 10 g portion was added to a PowerBead tubes containing homogenizing and lysis buffer, before gently vortexing to mix. Then, total genomic DNA was extracted using the PowerMax Soil DNA Isolation Kit (No. 12988-10, Qiagen), according to the manufacturer’s protocol. DNA was quantified using the Nano Drop ND-2000 Spectrophotometer (Thermofisher, Waltham, MA, USA).

### 4.3. RPA Primer and Probe Design

A specific *H. schachtii* DNA fragment (GenBank accession No. MW854319, accessed on 1 April 2021) was amplified using the random primer OPA06 (Jiang Chen et al., unpublished data). Three pairs of specific RPA primers were designed on the basis of a specific *H. schachtii* fragment using Primer Premier 6.0 (Table 1), One primer set (HsRPA-F2 and HsRPA-R2) was selected for further testing, and the resulting amplicon contained at least one protospacer adjacent motif (PAM) site (TTTA) for Cas12a recognition. The Hs-probe, located between the upstream and downstream primers, did not duplicate the primers, and a tetrahydrofuran (THF) probe was added as an Nfoase recognition site.

### 4.4. RPA Reaction

RPA reactions were conducted as previously described by Piepenburg et al. [13], using a kit (WLN8203KIT; AMP-Future Biotech Co. Ltd., Nanjing, China). The RPA amplification reaction contained 29.4 µL of Buffer A, 2.5 µL of Buffer B, 2 µL of each primer, 2 µL of template DNA, and double-distilled water (ddH_2_O) to 50 µL; reactions were incubated at 37 °C for 10–30 min.

### 4.5. Lateral Flow Dipstick (LFD) Assay

In the RPA/LFD assay, RPA reactions were performed as described above, with slight modifications, as follows: the 5′ end of the downstream HsRPA-R2 primer was labeled with biotin, and the 5′ end of the HsRPA-probe was labeled with FAM. After the RPA reaction, 10 µL of each reaction product was transferred into a 1.5 mL tube with 190 µL of ddH_2_O, mixed evenly, and then an LFD Strip (Hangzhou Youstar Biological Technology Co. Ltd., D003-3, Hangzhou, China) was inserted into the mixture. The results were observed after approximately 10 min by the naked eye. Positive results were indicated by two lines, including a test line and a control line, whereas only the control line was observed in the negative samples.

### 4.6. RPA/Cas12a Detection

For RPA with Cas12a, the detection method was as described previously. An ssDNA, composed of five consecutive T nucleotides, with the 5′ end conjugated to a FAM marker and the 3′ end to BHQ1 was designed according to a specific sequence. gRNA was designed on the basis of the PAM (TTTA) site of Cas12a (Table 1). The optimal parameters for 20 μL Cas12a reactions were as follows: 1.0 µL of Cas12a (NEB #M0653S, New England BioLabs, Ipswich, MA, USA), 2.0 µL of 10 × Cas12a reaction buffer, 0.4 µL of gRNA (3 µmol/L), 0.1 µL of ssDNA (10 µmol/L), 1 µL of RPA amplification product, and 15.5 µL of ddH_2_O. Fluorescence values generated by the reaction system were detected using a real-time fluorescence quantitative instrument (Applied Biosystems™ 7500, Waltham, MA, USA).

### 4.7. RPA Assay Specificity Test

To evaluate the specificity of the RPA method, DNA was isolated from 23 nematode populations (including nine *H. schachtii* and three *H. glycines* populations, as well as 11 other cyst nematodes species) and used as a template for RPA analysis. Distilled water was used for the template-free control (NTC) reaction. The specificity test was repeated three times for each population.

### 4.8. RPA Assay Sensitivity Test

To determine the sensitivity of RPA analysis, serial tenfold dilutions of SBCN genomic DNA (at an initial concentration of 100 ng/µL) were prepared in sterile distilled water, genomic DNA samples from a single cyst and a single female nematode were serially diluted in tenfold increments in sterile water, and a single *H. schachtii* J2 was serially diluted in fourfold increments in sterile water. The different dilutions of DNA were analyzed separately using the RPA method as described above. The sensitivity test was repeated three times for each experiment.

### 4.9. Direct Detection of Heterodera schachtii in Inoculated Plant Roots and Soil

To estimate the potential of RPA to detect *H. schachtii* directly in plants and root samples, two hosts of *H. schachtii,* sugar beets (cv. SD21816) and oilseed rape (cv. No. 11 Xinyou), were cultured in 10 cm diameter pots containing autoclaved soil in a greenhouse. Three weeks after transplanting, 500 *H. schachtii* J2s were inoculated per plant and incubated at 25 °C with a 16 h light/8 h dark cycle. Roots were collected at 3, 9, and 15 dai. Each sample was divided into two equal parts, one of which was used to count the presence of nematodes in the inoculated roots after staining with acid fuchsin, as described by Byrd et al. [51], while the other was stored at −80 °C for DNA extraction. In addition, 1, 5, 10, 15, and 20 *H. schachtii* J2s were placed into 10 g of autoclaved soil, with autoclaved soil alone serving as the negative control, and genomic DNA isolation and RPA detection were conducted described above. To ensure the accuracy of the results, three independent DNA extractions were performed for each experiment.

### 4.10. Detection of Heterodera schachtii in Natural Field Roots

To evaluate the ability of the RPA/LFD assay to detect *H. schachtii* in the field, 10 root samples were collected from Xinjiang, where beet cyst nematode was first reported in China, and five other field samples were collected from sugar beet-planting areas in Inner Mongolia and Hebei Provinces (China) (Table 3). Samples were collected in June 2020. DNA was directly extracted from the sugar beet roots and analyzed using the RPA method. To confirm the accuracy of the detection results, conventional PCR was conducted using the previously reported *H. schachtii*-specific primers, SHF6 and rDNA2 [8]. *H. schachtii* genomic DNA was used as a positive control and noninfected plants as a negative control. The test was repeated three times for each sample.

## Figures and Tables

**Table 1:** Codes and sequences of primers, probes, and gRNA used in this study.

Primers	Sequences	Purpose	References
HsRPA-F1	5′-GAGAATTGAATTTATCTCCTTTTCACCTCAGCTC-3′	RPA amplification	This study
HsRPA-R1	5′-CACAGAGACAACACGAAGGAGCGAGCTCAATG-3′		
HsRPA-F2	5′-TATCATCCAAATTTTGAGCGGTTCAAAGCG-3′		
HsRPA-R2	5′-GGCCTTTTGCGTACACAGCGAAACTTGCGAATC-3′		
HsRPA-F3	5′-CAACAACTACCACCACAATTACCGAACCACCG-3′		
HsRPA-R3	5′-CAACTCCTTCAATGATTTGTCTAAGAATTCTC-3′		
ssDNA	5′-FAMTTTTT-3′BHQ1	Cas12a degradation	
Hs-probe	5′-FAMCACCCGAGAATTGAATTTATCTCCTTTTCAC(THF)TCAGCTCGGCTATGTGTTG-3′		
Hs-gRNA	5′-AAUUUCUACUGUUGUAGAUGAAUUUAUCUCCUUUUCACCUCA-3′		
SHF6	5′-GTTCTTACGTTACTTCCA-3′	PCR amplification	[8]
rDNA2	5′-TTTCACTCGCCGTTACTAAGG-3′		

**Table 2:** Cyst nematode populations studied.

Species Code	Species	Population Origin	Host	Sampling Date	References
Hs1	*H. schachtii*	Germany	Sugar beet	- ^a^	[30]
Hs2	*H. schachtii*	Belgium	Sugar beet	- ^a^	[9]
Hs3	*H. schachtii*	Bozova, anliurfa Province, Turkey	Sugar beet	-	[31]
Hs4	*H. schachtii*	Karakprü anliurfa Province,Turkey	Sugar beet	-	[31]
Hs5	*H. schachtii*	Siverek, anliurfa Province, Turkey	Sugar beet	-	[31]
Hs6	*H. schachtii*	Xinyuan county Xinjiang, China	Sugar beet	Sept. 2019	This study
Hs7	*H. schachtii*	Xinyuan county Xinjiang, China	Sugar beet	Sept. 2019	This study
Hs8	*H. schachtii*	Xinyuan county Xinjiang, China	Sugar beet	Sept. 2019	This study
Hs9	*H. schachtii*	Emin county, Xinjiang, China	Sugar beet	Sept. 2019	This study
Hg1	*H. glycines*	Langfang, Hebei, China	Soybean	Oct. 2019	[32]
Hg2	*H. glycines*	Heilongjiang, China	Soybean	Oct. 2019	[32]
Hg3	*H. glycines*	Japan	-	- ^b^	This study
Hso	*H. sojae*	Jiangxi, China	Soybean	Sept. 2017	[33]
Hc	*H. cruciferae*	Hubei, China	Oilseed rape	Sept. 2017	This study
Ha	*H. avenae*	Norway	Wheat	Sept. 2016	[34]
Hf	*H. filipjevi*	Tangyin. Henan, China	Wheat	July 2019	[35]
Hl	*H. latinpones*	Belgium	Wheat	- ^a^	[36]
He	*H. elachista*	Hunan, China	Rice	Dec. 2016	[37]
Hh	*H. humuli*	Guizhou, China	*Weeds*	July 2018	This study
Hr	*H. ripae*	Guizhou, China	Weeds	July 2018	This study
Hz	*H. zeae*	Laibin, Guangxi, China	Maize	Mar. 2018	[38]
Gr	*Globodera rostochiensis*	Belgium	Potato	- ^a^	[39]
Cc	*Cactodera* *cacti*	Liaoning, China	Weeds	July 2018	[40]

- No record. ^a^ Samples supplied by Prof. Maurice Mones (Institute for Agriculture and Fisheries Research (ILVO), Belgium). ^b^ Sample intercepted in the soil from fruit trees imported from Japan by Beijing Customs.

**Table 3:** Detection of *H. schachtii* in natural field samples.

Samples	Host	Location	Cyst Nematode Density ^a^	Detection Results		
				RPA/LFD (Detections/Trials)	RPA/CRISPR (Detections/Trial)	PCR
1	Sugar beet	Huocheng County, Xinjiang, China	0	0/3	0/3	−
2		Zhaosu County, Xinjiang, China	0	0/3	0/3	−
3		Xinyuan county-1, Xinjiang, China	22 ± 7	3/3	3/3	+
4		Xinyuan county-2, Xinjiang, China	46 ± 4	3/3	3/3	+
5		Xinyuan county-3, Xinjiang, China	38 ± 5	3/3	3/3	+
6		Xinyuan county-4, Xinjiang, China	20 ± 5	3/3	3/3	+
7		Gongliu county, Xinjiang, China	0	0/3	0/3	−
8		Gongliu county, Xinjiang, China	0	0/3	0/3	−
9		Emin county, Xinjiang, China	11 ± 4	3/3	3/3	+
10		Emin county, Xinjiang, China	19 ± 4	3/3	3/3	+
11		Keyouqian Banner, Inner Mongolia, China	0	0/3	0/3	−
12		Urad Front Banner, Inner Mongolia, China	0	0/3	0/3	−
13		Linxi county, Inner Mongolia, China	0	0/3	0/3	−
14		Zhangbei county, Hebei province, China	5 ± 2	3/3	3/3	+
15		Kangbao county, Hebei province, China	0	0/3	0/3	−

^a^ Numbers of *Heterodera* spp. were counted after staining with acid fuchsin in 0.2 of g sugar beet. + Indicates the presence of the *H. schachtii*-specific bands; − indicates the absence of the *H. schachtii*-specific bands.

## Data Availability

The original contributions presented in the study are included in the article. Further inquiries can be directed to the corresponding author.

## Supplementary Materials

The following are available online at https://www.mdpi.com/article/10.3390/ijms222212577/s1.
