# Supplementary material for: Rapid and Visual Detection of Heterodera schachtii Using Recombinase Polymerase Amplification Combined with Cas12a-Mediated Technology
Source: Int J Mol Sci. 2021 Nov 22;22(22):12577. doi: 10.3390/ijms222212577 (PMC8618885; doi:10.3390/ijms222212577)
Supplement: Supplementary file 1 [file ijms-22-12577-s001.zip › ijms-1446312-supplementary.pdf]

Supplementary Material

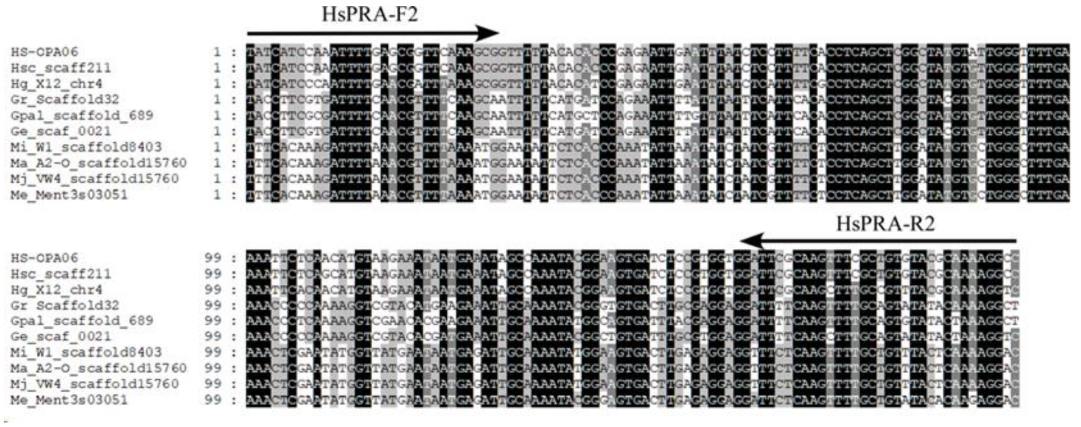

Figure S1. This sequences alignment results between *Heterodera schachtii* and other homologous nematodes. HS-OPA06: *H. schachtii* (GenBank accession No. MW854319, accessed on 1 April 2021); Hsc_scaff211: *H. schachtii* (*JAHGVF010000211.1*); Hg_X12_chr4: *H. glycines* (*VAPQ01000257.1*); Gr_Scaffold32: *G. rostochiensis* (*JAEVLO010000032.1*); Gpal_scaffold_689: *G. pallida* (*CBXT010012390.1*); Ge_scaf_0021: *G. ellingtonae* (*MEIZ01000021.1*); Mi_W1_scaffold8403: *M. incognita* (*RCFL01002996.1*); Ma_A2-0_scaffold15760: *M. incognita* (*RCFL01002996.1*); Mj_VW4_scaffold15760: *M. javanica* (*RCFK01013271.1*); Me_Ment3s03051: *M. enterolobii* (*CAJEWN010003051.1*).
